# Prediction of pharmacological treatment efficacy using electroencephalography-based salience network in patients with major depressive disorder

**DOI:** 10.3389/fpsyt.2024.1469645

**Published:** 2024-10-17

**Authors:** Kang-Min Choi, Taegyeong Lee, Chang-Hwan Im, Seung-Hwan Lee

**Affiliations:** ^1^ Clinical Emotion and Cognition Research Laboratory, Inje University, Goyang, Republic of Korea; ^2^ School of Electronic Engineering, Hanyang University, Seoul, Republic of Korea; ^3^ Department of Biomedical Engineering, Hanyang University, Seoul, Republic of Korea; ^4^ Department of Psychiatry, Ilsan Paik Hospital, Inje University College of Medicine, Goyang, Republic of Korea; ^5^ Bwave Inc, Goyang, Republic of Korea

**Keywords:** electroencephalography, major depressive disorder, salience network, prediction of antidepressant responsiveness, condition-dependent functional network

## Abstract

**Introduction:**

Recent resting-state electroencephalogram (EEG) studies have consistently reported an association between aberrant functional brain networks (FBNs) and treatment-resistant traits in patients with major depressive disorder (MDD). However, little is known about the changes in FBNs in response to external stimuli in these patients. This study investigates whether changes in the salience network (SN) could predict responsiveness to pharmacological treatment in resting-state and external stimuli conditions.

**Methods:**

Thirty-one drug-naïve patients with MDD (aged 46.61 ± 10.05, female 28) and twenty-one healthy controls (aged 43.86 ± 14.14, female 19) participated in the study. After 8 weeks of pharmacological treatment, the patients were divided into non-remitted MDD (nrMDD, n = 14) and remitted-MDD (rMDD, n = 17) groups. EEG data under three conditions (resting-state, standard, and deviant) were analyzed. The SN was constructed with three cortical regions as nodes and weighted phase-lag index as edges, across alpha, low-beta, high-beta, and gamma bands. A repeated measures analysis of the variance model was used to examine the group-by-condition interaction. Machine learning-based classification analyses were also conducted between the nrMDD and rMDD groups.

**Results:**

A notable group-by-condition interaction was observed in the high-beta band between nrMDD and rMDD. Specifically, patients with nrMDD exhibited hypoconnectivity between the dorsal anterior cingulate cortex and right insula (p = 0.030). The classification analysis yielded a maximum classification accuracy of 80.65%.

**Conclusion:**

Our study suggests that abnormal condition-dependent changes in the SN could serve as potential predictors of pharmacological treatment efficacy in patients with MDD.

## 1 Introduction

Major depressive disorder (MDD) is a prevalent yet heterogeneous mental disorder. It is widely known that about 30% of patients do not respond to antidepressant treatment even though it is one of the most popular and neurobiologically validated therapies for MDD (1–3). Predicting the efficacy of antidepressant treatment is a crucial issue for personalized therapy, aiming to avoid ineffective treatment so that minimize unwarranted side effects resulting from ineffective medications (3, 4).

For the prediction of the treatment response in patients with MDD, a variety of neuroimaging studies have focused on the identification of reliable biomarkers. Recently, numerous studies have consistently reported that patients who exhibit similar functional brain network (FBN) patterns to healthy controls (HCs) tend to show a strong response to antidepressant treatment (5, 6). Specifically, several studies have suggested that aberrant resting-state functional connectivity (FC) patterns could serve as effective predictors of treatment outcomes in patients with MDD. In recent years, these FC patterns have been utilized as features to train machine-learning models, enhancing the performance in predicting treatment response.

Among various neuroimaging modalities, electroencephalography (EEG) is advantageous for studying FBN due to its great temporal resolution and cost-effectiveness (4, 7, 8). Some studies found distinct resting-state FBN patterns in patients with medication treatment-resistant MDD. For example, Whitton et al. (9) revealed that resting-state theta-band functional connectivity between the rostral anterior cingulate cortex and right anterior insula was associated with the efficacy of the antidepressant. Using an unsupervised machine learning (ML) model, Zhang et al. (6) successfully divided patients with MDD and post-traumatic stress disorder (PTSD) into two subtypes: drug responders and resistors. Relatively fewer EEG studies identified distinct FBN patterns in these patients under conditions involving external stimulation. For example, Sumner et al. (10) reported that rapid antidepressant efficacy was associated with dynamic forward connectivity in response to the unexpected auditory stimuli between the right primary auditory cortex and the right inferior temporal cortex. Overall, most EEG studies have primarily concentrated on investigating a single paradigm FBN pattern, particularly in the context of the resting-state condition.

Several up-to-date neuroimaging studies have investigated various condition-dependent brain activities to explore dysfunctional pathophysiological pathways (11–17). Among them, recent studies have consistently suggested that our understanding of neurobiology and various mental disorders could be broadened by investigating condition-dependent FBN patterns, including stimuli-based FBN patterns themselves and comparison of FBN patterns for various conditions (e.g., resting vs. stimuli, target vs. non-target) (11–14). However, it is yet to be investigated whether the condition-dependent changes in EEG-FBN could predict the treatment response in patients with MDD, despite their significant potential. For example, several EEG studies found that patients with drug-resistant MDD exhibited malfunctioning salience network (SN) connectivity patterns in the resting state, known for involvement of the selective attention control by processing salient events (18–22). Considering the role of SN, it is reasonable to hypothesize that those patients would also show abnormal FBN patterns under the condition with external salient stimulation. The malfunctioning changes in stimuli-induced SN have been observed in patients with treatment-resistant MDD in functional magnetic resonance imaging (fMRI) studies (12, 14, 20, 23).

In this study, we investigated condition-dependent changes in EEG-derived FBN in patients with MDD, using a dual-paradigm consisting of resting state and passive auditory oddball paradigm, generally known as the mismatch negativity (MMN) paradigms. Specifically, the SN was explored between patients with non-remitted MDD (nrMDD) and those with remitted MDD (rMDD) after an 8-week pharmaceutical therapy. The study is based on the hypothesis that the condition-dependent SN would show distinct patterns between groups; particularly, patients with nrMDD would exhibit more divergent patterns compared to demographically-matched healthy controls (HCs), consistent with existing resting-state FBN studies. To demonstrate the potential of the condition-dependent changes in SN as predictors of antidepressant responsiveness, we performed statistical analysis and ML-based classification analysis.

## 2 Methods and materials

### 2.1 Participants

A total of 33 patients with MDD (aged 46.00 ± 10.04, male: 3) and 22 HCs (aged 44.36 ± 14.00, male: 3) participated in the study. Due to poor data quality, the data of two patients with MDD and one HC were discarded in the subsequent analysis; hence, data analysis was performed with 31 patients with MDD (aged 46.61 ± 10.05, male: 3) and 21 healthy controls (aged 43.86 ± 14.14, male: 2).

Patients with MDD were recruited from the Department of Psychiatry at the Inje University Ilsan Paik Hospital. The MDD was diagnosed by board-certified psychiatrists, based on the Structured Clinical Interview for the Diagnostic and Statistical Manual of Mental Disorders, 5th edition (APA). The patients had no history of neurological illness, intellectual disability, substance abuse, head injury, or impaired hearing ability. Patients did not take any medication for at least one month before the study. After data acquisition, they received vortioxetine 10 mg po for the first week, followed by 20 mg po for the second week. Subsequently, the dosage was maintained flexibly ranging from 10 to 20 mg po, until the conclusion of the treatment period (i.e., 8^th^ week). Concerning the depressive symptom severity at the conclusion, namely, Hamilton Depression (Ham-D) Rating Scale score for the 8th week (Ham-D_8_) (details in the following section) patients were finally divided into two groups: (i) non-remitted MDD (nrMDD; Ham-D_8_ ≥ 8, *n* = 14), and (ii) remitted MDD (rMDD; Ham-D_8_ < 8, *n* = 17).

HCs were recruited from the community using flyers and posters. They also had no history of head injury or medications with psychiatric disorders, and also have no family history of psychiatric disorders. All the participants signed an informed consent form approved by the Institutional Review Board at Inje University Ilsan Paik Hospital before participation in the experiment (IRB No. 2016-08-017).

### 2.2 Symptomatic and psychological measures

The symptom severity of depression and anxiety were assessed by the Hamilton Depression Rating Scale (Ham-D) (24), and Hamilton Anxiety (Ham-A) (25) rating scales, respectively. The Ham-D and Ham-A consisted of 17 and 14 items, respectively. After 8 weeks of treatment, patients with a Ham-D score lower than 8 were classified as remitted MDD (rMDD), while the others were categorized as non-remitted MDD (nrMDD). The Ham-D and Ham-A were acquired at the 0th, 2nd, 4th, and 8th weeks (**Supplementary Table S1**). Only Ham-D and Ham-A were utilized from our previous study, as other measures were not of interest in the current study.

### 2.3 Experimental conditions

All participants engaged in two experimental paradigms: (i) resting-state (RS), and (ii) MMN paradigms. In the RS paradigm, participants closed their eyes for 5 min without any stimulation. Then, a duration-variant auditory oddball paradigm was conducted. The probability of deviant stimulus occurrence was set to 10% in a total of 750 trials. Participants were required to watch a silent movie during the auditory stimulus presentation and instructed not to focus on the auditory stimuli.

In the passive oddball experiment, the auditory stimuli were delivered binaurally with noise-canceling MDR-D777 headphones (Sony, Tokyo, Japan). The loudness and the pitch of all stimuli were set to 85 dB and 1000 Hz, respectively. The duration of the stimulation was set to 50 ms for the standard stimuli (Std) but 100 ms for the deviant stimuli (Dev), with 10 ms of rising and falling edges. The interstimulus interval was fixed at 600 ms.

### 2.4 Signal acquisition and pre-processing

The participants were asked to sit comfortably in a chair. Biosignal data were acquired using Neuroscan SynAmps2 (Compumedics USA, El Paso, TX, USA). For the EEG, a total of 64 Ag-AgCl electrodes mounted on a Quik-Cap were placed following the extended 10-20 system. For the electrooculogram (EOG), four electrodes were placed above and below the left eye and on the outer canthi of both eyes. Throughout signal acquisition, the impedance of all the electrodes was below 5 kΩ. The signals were recorded at 1,000 Hz of sampling rate and then bandpass filtered between 0.1 - 100 Hz.

The acquired signals were pre-processed using the EEGLAB toolbox (26) implemented in MATLAB R2019b (MathWorks, Natick, MA, USA). For the elimination of physiological artifacts, independent component analysis was performed. The components containing artifacts including EOG, electromyogram, and electrocardiogram were manually rejected. The EEG signals were then band-pass filtered between 0.1 – 50 Hz using a 6th-order Butterworth filter. After manual inspection, the EEG signals were segmented into 700 ms. For the auditory oddball data, the epochs ranged from 100 ms of a pre-stimulus interval to 600 ms of a post-stimulus interval (i.e., -100 – 600 ms). The segmented data were detrended and then baseline corrected using the pre-stimulus interval data. For the resting-state data, the epochs were segmented using the same length of time window (i.e., 700 ms) without any overlap. Regardless of experimental paradigms, all epochs with absolute maximum values exceeding 75 μV were excluded from the analysis. Among the noise-free segments, 250, 300, and 45 epochs were randomly selected for the RS, Std, and Dev conditions, respectively, from each participant.

### 2.5 Construction of salience network

For the construction of the SN, source localization was performed using the Brainstorm toolbox (27). The source activities were calculated with a depth-weighted L2-norm estimator from the randomly segmented EEG signals. Excluding mastoid electrodes, we selected all 62 EEG electrodes for source localization. The Colin27 MRI brain template with 15,002 voxels was employed for the estimation of the cortical activities. For the construction of the lead field matrix, a three-layer boundary element model was implemented from the OpenMEEG project software (28).

Three regions of interest (ROIs) were selected as the representative nodes of the SN according to the previous fMRI studies: (i) dorsal anterior cingulate cortex (dACC); (ii) left insula (lIns); (iii) right insula (rIns) (**Supplementary Materials**). The Montreal Neurological Institute (MNI) coordinates of the ROI seeds were determined as centers of gravity of the provided coordinates (**Supplementary Table S2**), with manual verification of the coordinates. From the seed coordinates, the voxels within a 5 mm Euclidean distance were selected as representative ones. Finally, the representative source signal of the ROIs was obtained by the first component of the principal component analysis, using source signals acquired from the neighboring voxels.

The weighted phase-lag index (wPLI) (29) was calculated for evaluation of the edge between a pair of nodes (i.e., FC) for 4 frequency bands: (i) alpha (8 – 12 Hz); (ii) low beta (12 – 18 Hz); (iii) high beta (18 – 30 Hz); (iv) gamma (30 – 50 Hz). For each 0.7 s epoch, a pair of the representative source signals from the ROIs were bandpass filtered according to the frequency band. Subsequently, the Hilbert transform-based instantaneous phase was calculated. Finally, the absolute value of the temporal expectation of the instantaneous phase difference between the ROIs was divided by the temporal expectation of the absolute phase difference, as follows (30):


$$wPLI=\frac{\left|E\left(\sin\Delta \phi \left(t\right)\right)\right|}{E\left(\left|\sin\Delta \phi \left(t\right)\right|\right)}$$

where the *Δϕ(t)* denotes the difference in instantaneous phase as a function of time, t, |.| denotes the absolute operator, and the E(.) denotes the expectation operator across the time. Herein, the phase differences of the intervals for the initial and end 0.1 s were excluded from the calculation of the expectation values to eliminate edge effects caused by the filtering and Hilbert transform, as well as discard the baseline interval data in the oddball paradigm. The wPLI values can vary from 0 (entirely out-of-phase) to 1 (entirely phase-locked). It should be noted that the wPLI values were calculated for each band (i.e., *n* = 4), pair of nodes (*n* = 3), and epoch (n = 250, 300, and 45 for RS, Std, and Dev, respectively), and subsequently averaged across epochs. Finally, the FCs were defined as these averaged wPLI values. In addition, the global strength of the SN was evaluated as the sum of all pairs of the wPLI values (i.e., 3 wPLI values).

### 2.6 Statistical analysis

For verification of the assumption of data normality, skewness and kurtosis of the data distribution were examined. All absolute values of the skewness and kurtosis were less than 2 and 7, respectively (31); hence, all the data distributions were assumed to follow a normal distribution. For comparison of the demographic differences between 3 groups (i.e., nrMDD, rMDD, and HC), an analysis of variance (ANOVA) was used for age and education, while the chi-squared test was used for the sex ratio.

For evaluation of the group-by-condition interaction in the MDD groups, repeated-measures ANOVA (rmANOVA) was performed for three experimental conditions (i.e., RS, Std, Dev) as within-subject factors and the group (nrMDD vs. rMDD) as the between-subject factors, for each frequency band. We initially tested global strength and subsequently tested the three pairs of wPLIs if notable group-related effects were observed. Regarding rmANOVA, Mauchly’s sphericity assumption was used given that the data distribution met the condition; otherwise, Greenhouse-Geisser correction was alternatively used. When significant group-related interaction was observed, *post-hoc* analyses were performed as follows. First, rmANOVA was performed for each group. Second, an independent t-test was performed. To avoid multiple correction issues, the bootstrap resampling technique (*n* = 5,000) was performed (32).

### 2.7 Feature ext

To demonstrate the potential of condition-dependent changes in SN patterns to predict pharmacological treatment response in patients with MDD, a further ML-based classification analysis was conducted. Consequently, classification between the MDD groups (nrMDD vs. rMDD) was performed using EEG features.

#### 2.7.1 Feature extraction

From the SN-related measures, two types of condition-dependent FCs were determined as feature candidates. First, three pairs of FCs in the Dev-condition were selected. Second, three pairs of FC differences were selected, by subtracting FC values in the Std condition from FC values in the Dev condition, similar to the traditional MMN amplitude.

Some conventional measures were also included as feature candidates to enhance the classification performance. From the RS condition, absolute band power was calculated over the six cortical regions: bilateral frontal, central, and parieto-occipital areas. In addition, MMN amplitude was obtained from the frontocentral cortical regions. To obtain the MMN amplitude for each participant, the difference ERP curve was acquired by subtracting the Std-ERP curve from the Dev-ERP curve. Both ERP curves were obtained by averaging epochs for each condition, with bandpass filtered at 0.1 – 30 Hz using the 6th-order Butterworth filter. The potential values lasting from 130 ms to 280 ms were averaged and then defined as MMN amplitude. For more detail, please refer to our previous study (33).

#### 2.7.2 Cross-validation and feature selection

To assess the performance of the classifiers, leave-one-out cross-validation (LOOCV) was conducted. Subsequently, the optimal feature subset was determined from the training dataset using the Fisher score (34). The number of selected features ranged from 1 to 15, the Fisher scores of which were the highest, to prevent the dimensionality-related overfitting issue. The selected features were then normalized to z-score to eliminate the inter-feature biases. It is noted that the statistics used for normalization (i.e., mean and standard deviation) were extracted from the training datasets to prevent information leakage.

#### 2.7.3 Classification analysis

For the classification analysis, four ML-based classifiers were utilized to differentiate between nrMDD and rMDD: linear discriminant analysis (LDA), support vector machine (SVM), k-nearest neighbors (KNN), and naive-Bayes (NB). To evaluate the classification performance, three indices were computed: (i) classification accuracy, (ii) sensitivity, and (iii) specificity. Specifically, sensitivity and specificity were determined using nrMDD as the reference group. For instance, sensitivity was defined as the proportion of patients with nrMDD who were correctly classified. Finally, the receiver operating characteristic (ROC) curve was generated by using various decision thresholds, for the best-performing classifier. From the ROC curve, the area under the curve (AUC) was calculated for the evaluation of the performance of the classifier.

## 3 Results

### 3.1 Demographic and psychological measures

No significant demographic differences between the nrMDD, rMDD, and HC groups (*p* > 0.1 for all variables; **Table 1**). Furthermore, no significant differences were found in terms of baseline symptom severity (i.e., Ham-D and Ham-A; *p* > 0.1).

**Table 1:** Demography, symptom severity, and socio-cognitive function.

	nrMDD (*n* = 14)	rMDD (*n* = 17)	HC (*n* = 21)	*p-value*
Age	43.14 ± 11.07	48.35 ± 9.00	43.86 ± 14.14	*0.159*
Sex (M/F)	1/13	2/15	2/19	*0.764*
Education	13.86 ± 2.98	13.53 ± 3.43	13.24 ± 4.16	*0.778*
Ham-D				
Week 0	30.00 ± 5.57	26.24 ± 6.81		*0.108*
Week 8	17.14 ± 8.05	4.41± 1.77		*< 0.001*
Ham-A				
Week 0	27.07 ± 6.73	24.76 ± 6.57		*0.344*
Week 8	16.43 ± 7.36	4.06 ± 2.73		*< 0.001*

### 3.2 Comparison of the condition-dependent changes in SN patterns

**Figure 1:**
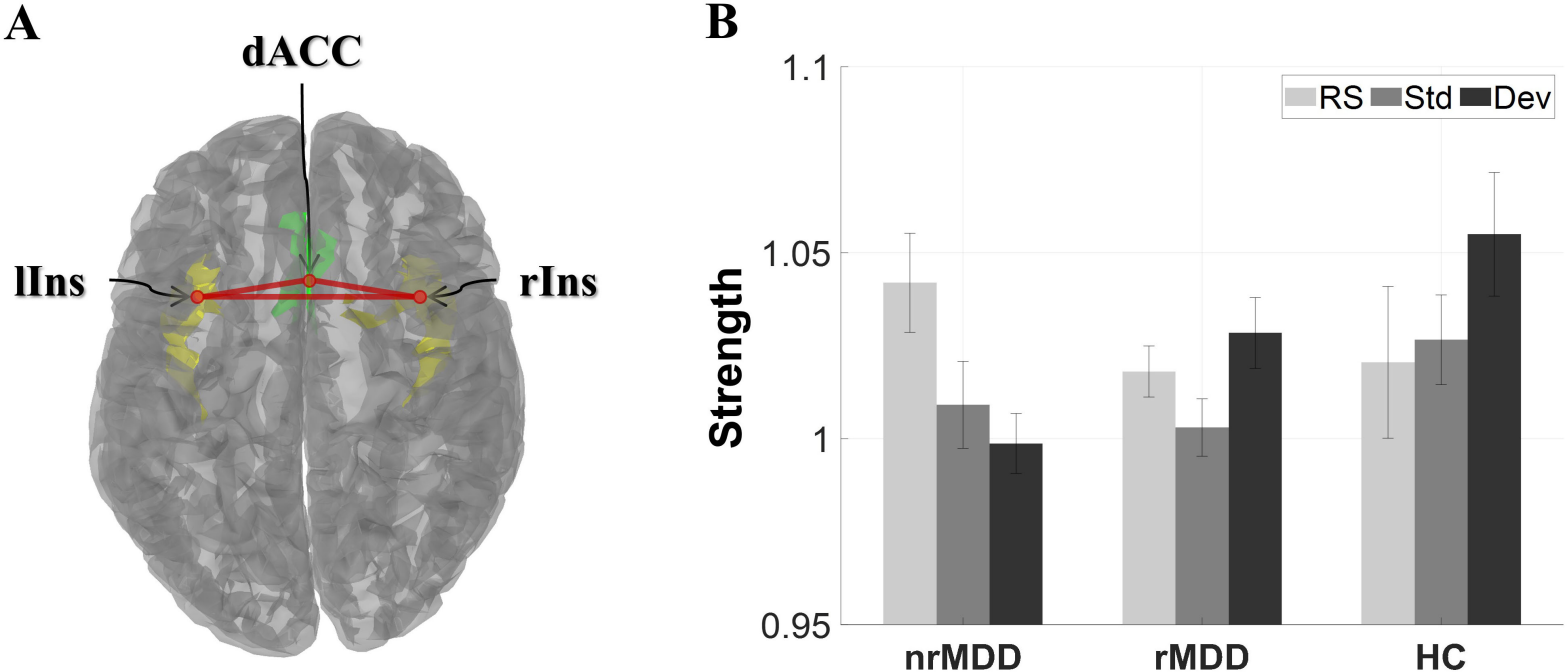
The global strength of the high-beta band salience network for each group under three different conditions. **(A)** Structure of the salience network, consisting of 3 regions of interest. **(B)** Global strength. The error-bars indicate the standard errors. dACC, dorsal anterior cingulate cortex; lIns, left insula; rIns, right insula; nrMDD (*n* = 14), non-remitted MDD; rMDD (*n* = 17), remitted MDD; HC (*n* = 21), healthy control. The brain image was obtained from the Brainstorm toolbox.

In qualitative terms, patients with nrMDD exhibited aberrant patterns of condition-dependent changes in the high-beta band SN, demonstrating an opposite trend compared to HC. More specifically, while transitioning from RS- to Std- and Dev-condition, HC showed an increasing tendency in SN strength, whereas patients with nrMDD showed a decreasing tendency (**Figure 1**). Unlike patients with nrMDD, those with rMDD showed relatively similar condition-dependent changing patterns compared to HC.

In terms of SN strength, there was a notable group-by-condition interaction between nrMDD and rMDD in the high-beta band; however, it did not reach the significant level (*p* = 0.066; **Figure 1**). However, there was no other significant group-related effect.

**Figure 2:**
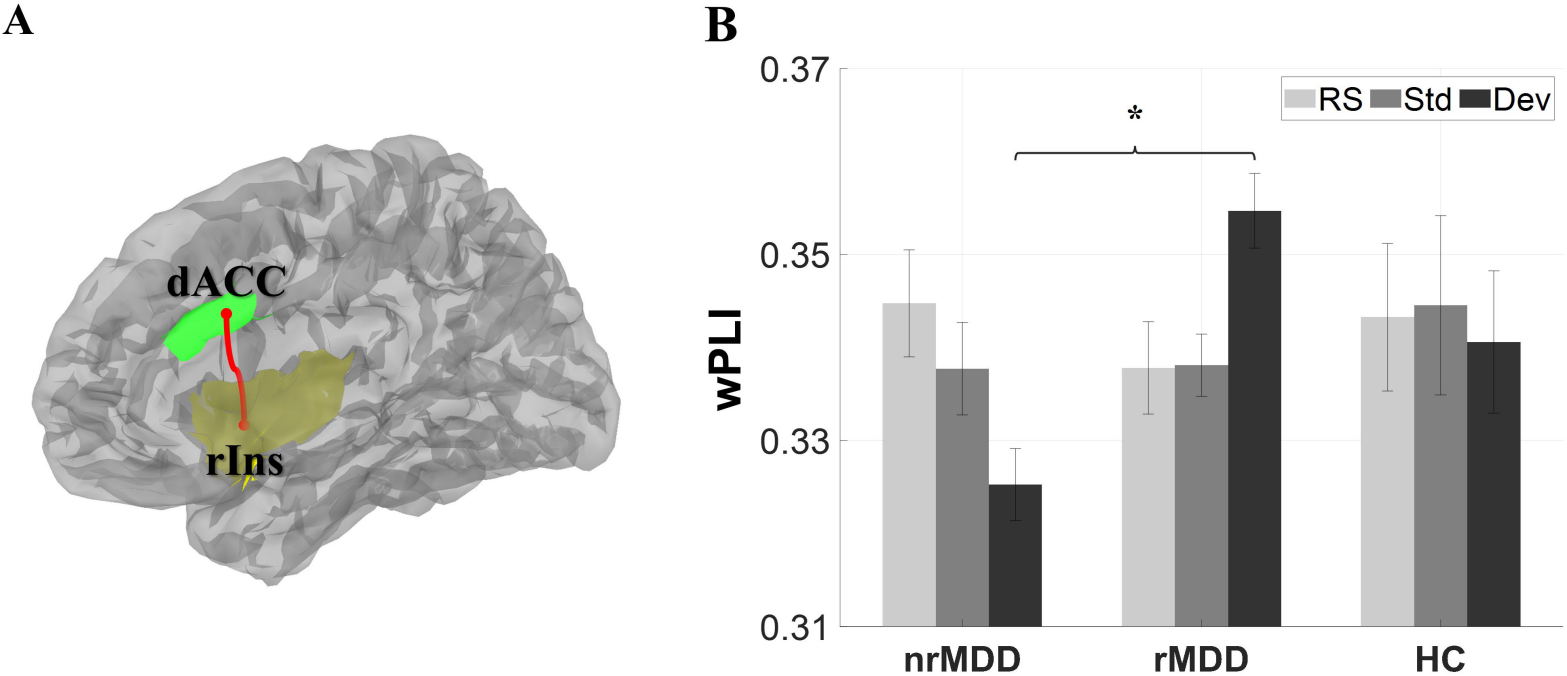
Functional connectivity (FC) between the dorsal anterior cingulate cortex (dACC) and right insula (rIns). **(A)** Structure of the dACC and rIns. **(B)** FC. The error-bars indicate the standard errors. *p < 0.05. dACC, dorsal anterior cingulate cortex; rIns, right insula; nrMDD (*n* = 14), non-remitted MDD; rMDD (*n* = 17), remitted MDD; HC (*n* = 21), healthy control. The brain image was obtained from the Brainstorm toolbox.

In the FC analysis, there was a significant group-by-condition interaction between nrMDD and rMDD in the high-beta band (*p* = 0.026; **Figure 2**). A *post-hoc* analysis revealed that nrMDD showed lower FC than rMDD under the Dev-condition (*p* = 0.030; 95%CI -0.055 ~ -0.005). However, there was no other significant group-related effect.

### 3.3 Classification analysis

**Figure 3:**
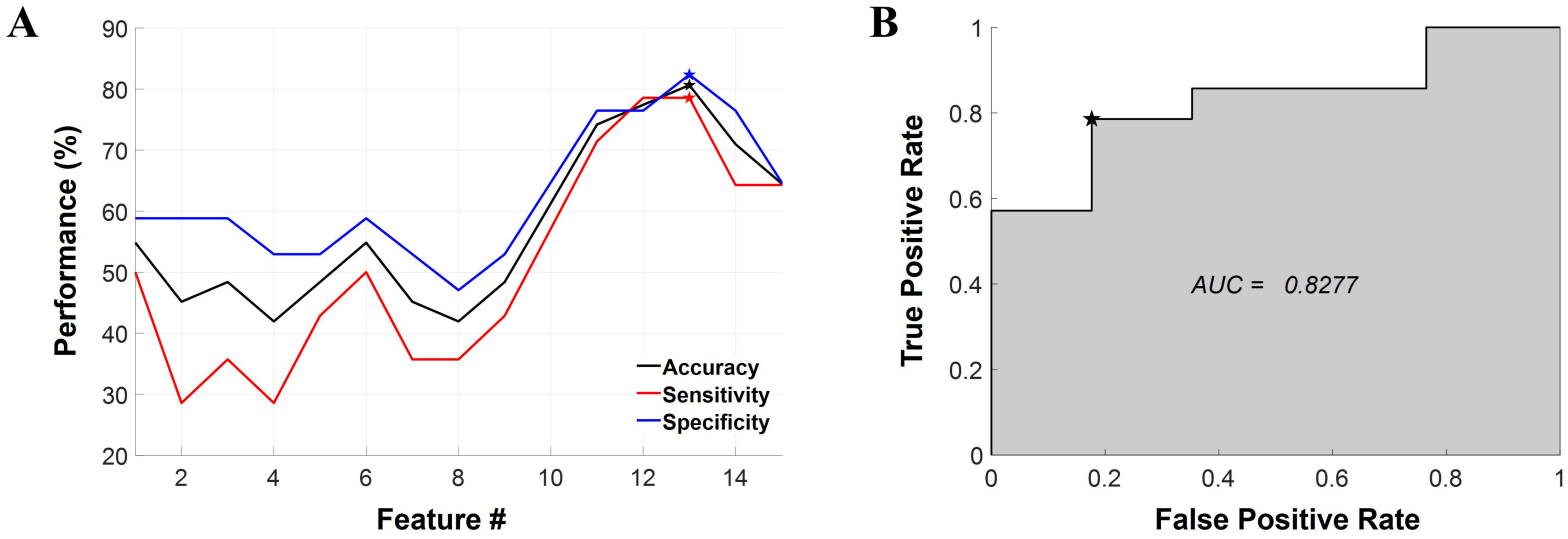
Results of the machine learning-based classification analysis. **(A)** The classification performance represented as a function of the number of features. The LDA model achieved optimal performance when 13 features were selected, as denoted by the pentagonal star symbol in the graph. Classification accuracy, sensitivity, and specificity are represented in black, red, and blue respectively. **(B)** The ROC curve for the best-performing classifier. The AUC is also provided within the graph. The chosen threshold on the ROC curve is marked by a pentagonal star symbol. LDA, linear discriminant analysis; ROC, receiver operating characteristic; AUC, area under the curve, Feature #, the number of features.

In the ML-based classification analysis, the best performance was yielded using an LDA classifier with 13 selected features (**Table 2**). The classification accuracy, sensitivity, and specificity values of the model were 80.65%, 78.57%, and 82.35%, respectively (**Figure 3A**). In addition, the AUC of the model was 0.8277 (**Figure 3B**). The model incorporated a variety of features, including FC under Dev-condition and conventional features (i.e., MMN and resting-state band power).

**Table 2:** The feature subset with the best performance (i.e., *n* = 13).

Feature	Frequency
MMN	31
FCdiff_rIns_dACC	31
FCdev_rIns_dACC	31
BPb2_LF	31
BPg_LC	31
BPg_LPO	31
FCdiff_lIns_dACC	30
BPg_RPO	30
BPb2_RF	28
BPg_RF	28
BPb2_RPO	27
BPg_LF	22
FCdev_lIns_rIns	14

MMN, mismatch negativity; FC, functional connectivity; FCdev, FC under the deviant condition; FCdiff, the difference between FCdev and FCstd; BP, band power; BPb2, high-beta BP; BPg, gamma BP; lIns, left insula; rIns, right insula; dACC, dorsal anterior cingulate cortex; L, left; R, right; F, frontal; C, central; PO, parieto-occipital.

## 4 Discussion

In this study, we investigated the condition-dependent changes in SN in patients with drug-naive nrMDD and rMDD using EEG. Our findings point to the high-beta band SN as a key condition-dependent network for predicting the efficacy of pharmacological treatments in patients with MDD. Specifically, the strength of SN displayed a contrasting condition-dependent tendency in patients with nrMDD compared to that of the HC group. In the deviant-stimulus condition, high-beta band FC between dACC and rIns exhibited an abnormal decrease in patients with nrMDD compared to those with rMDD. The ability of these condition-dependent SN-related features to serve as potential biomarkers for predicting responsiveness to antidepressants was further demonstrated through a machine learning (ML)-based classification analysis.

Our findings indicate that EEG-derived condition-dependent changes in FBN patterns could be reliable measures to predict the efficacy of pharmacological treatment. To the best of our knowledge, this is the first study to explore the pharmacological treatment response in patients with MDD using condition-dependent changes in FBN. To date, most EEG-derived FBN studies aiming for the prediction of treatment effects have been interested in resting-state FBN. It appears that patients with MDD showing similar resting-state FBN patterns to HC are more receptive to the pharmacological treatment effect (5, 6), than other neuroimaging modality-derived FBN studies (20, 35). However, despite their potential, little is known about the association between stimuli-related FBN patterns and treatment responsiveness. Recent neuroimaging studies have shown that the integration of stimuli-related and resting-state neural activity could facilitate a more comprehensive understanding of various psychiatric disorders (11–14). Specifically, our findings show that stimuli-related FBN patterns in patients with rMDD are relatively similar to those in HC, consistent with the resting-state FBN patterns. Therefore, stimuli-related FBN patterns might be interpreted as similar to the resting-state FBN patterns, underpinning their reliability.

Our results indicate that high-beta band SN is a key FBN exhibiting different condition-dependent FBN patterns between nrMDD and rMDD under the resting state and MMN paradigms. This is consistent with the previous MDD studies. Several FBN studies reported hyperconnectivity in the resting-state high-beta band for MDD (36, 37). Furthermore, several studies revealed that magnetic seizure therapy could help the hyperactive beta band be reduced to become normalized in patients with MDD (38, 39). Our findings indicate that the observed phenomena are more likely attributable to patients with treatment-resistant MDD. We also found significant group (nrMDD and rMDD)-by-interaction in the total-beta band (12 – 30 Hz; **Supplementary Material**), underpinning the suggestion. Furthermore, our findings bolster the view that a hyperactive resting-state SN in the high-beta band could lead to inefficient condition-dependent reconfiguration. It is worth mentioning that, despite its potential significance, theta band was excluded in the current study (9). This decision was made due to the limited time window resulting from the short inter-stimulus interval (0.6 s), which allows for at most 2.4 cycles of the 4-Hz oscillation, generally the lower limit of the theta band. Therefore, further studies are needed to investigate whether the theta-band SN could serve as a biomarker to predict antidepressant responsiveness in patients with MDD.

Our study suggests that patients with nrMDD are characterized by more dysfunctioning condition-dependent changes in SN. This finding is in line with the previous neuroimaging studies. Recent fMRI studies have consistently reported inefficient information transfer within the SN among patients with treatment-refractory MDD (20, 21). Such patients may experience a reduced quality of life due to diminished affective functions (40, 41), a key role of the SN. It is worth noting that our study also suggests that SN is readily reconfigured by the neutral-valence stimuli, demonstrated by condition-dependent changes in SN for HCs: strength of the high-beta band increased but that of the alpha band decreased under the stimulus condition, particularly for the deviant stimulation (**Supplementary Figure S1**). Beta-band phase synchronization is generally believed to be associated with attentional control and short-term working memory, by interacting with relatively distant regions (42, 43), providing support for our hypothesis.

Within the high-beta band SN, patients with nrMDD showed decreased FC between the dACC and rIns, compared to those with rMDD, which serves as a potential biomarker for predicting antidepressant response. Furthermore, sensitive condition-dependent change in FC between them was associated with the early period antidepressant responsiveness (**Supplementary Figure S2**). Both regions are well known to play essential roles in condition-dependent FBN reconfiguration. The rIns plays a role in selective attention by switching the attentional focus between the default mode network and the central executive network, according to the salient external stimulation (19, 44). dACC is a crucial hub for flexible FBN reconfiguration, the malfunctioning of which has been repetitively reported in MDD studies (45, 46). In conclusion, the hypoconnectivity between the dACC and rIns under the Dev condition in patients with nrMDD could be linked to the dysfunctions of the dynamic FBN flexibility, hindering efficient selective attention.

Based on the machine learning models, we showed the potential that the condition-dependent FBN characteristics identified in our study could serve as informative biomarkers to predict pharmacological treatment responsiveness. The optimal feature subset included various condition-dependent FBN patterns (i.e., strength and FCs) as well as various conventional measures (i.e., MMN and band powers). Our findings suggest that neurobiologically meaningful measures, derived from conventional experimental paradigms, can reflect condition-dependent changes in SN and have the potential to enhance the performance of machine learning classifiers as predictors. Notably, we acquired similar levels of sensitivity and specificity across various classifiers (**Supplementary Table S2**), including the best-performing classifier (**Figure 3**), rendering our results more reliable.

Our study has several limitations. Firstly, more replications are needed for our results to be generalizable, due to our small sample size and the lack of performance evaluation with an external dataset. Secondly, this study only considered an 8-week remission period for patients, without addressing other prognostic factors such as potential relapse. Thirdly, our study design did not include a placebo control group. Fourth, the majority of participants in the study were female, which may limit generalizability. This gender imbalance could be attributed to the higher prevalence of MDD in females and the lower participation rate of male patients in research studies. Finally, as individual brain MRI scans were not available in this study, a common template was used for estimating source estimation, which may have reduced the accuracy of estimating cortical electrophysiological activity. Future research will benefit from replicating these findings with a larger sample size and an external cohort to enhance generalizability. Additionally, examining an effective brain network or constructing a whole-brain network could provide meaningful insights into the underlying brain mechanisms in patients with non-remitted MDD.

Our study investigated the potential of condition-dependent changes in the EEG-derived salience network to predict antidepressant responsiveness in patients with MDD, assessed through both resting state and MMN paradigms. Patients with non-remitted MDD exhibited hyperconnectivity in the resting state but hypoconnectivity in response to salient stimuli (i.e., deviant condition) in the high-beta band SN, particularly for the FC between the dACC and rIns. In conclusion, understanding these condition-dependent connectivity patterns may contribute to the development of more targeted and effective treatments for MDD patients. It is hoped that our study pioneers research into condition-dependent changes in FBN.

## Data Availability

The data analyzed in this study is subject to the following licenses/restrictions: data are not publicly open because our dataset includes personal information. Requests to access these datasets should be directed to Seung-Hwan Lee, lshpss@paik.ac.kr.
