# Supplementary material for: Prediction of pharmacological treatment efficacy using electroencephalography-based salience network in patients with major depressive disorder
Source: Front Psychiatry. 2024 Oct 17;15:1469645. doi: 10.3389/fpsyt.2024.1469645 (PMC11525785; doi:10.3389/fpsyt.2024.1469645)
Supplement: Supplementary file 1 [file DataSheet1.docx]

Supplementary Material

# Condition-dependent changes in alpha band SN strength

## Alpha band

For MDD, there was a significant condition effect in the alpha band SN strength (*p* < 0.001, Figure S1). A post-hoc analysis revealed that the SN strength decreased in the Std-condition and the Dev-condition, as compared to the RS-condition (RS: 1.936 vs. Std: 1.742, *p* < 0.001; RS. 1.936 vs. Dev: 1.774, *p* < 0.001). However, there was no significant group-related effect.

Similarly, for HC, a significant condition effect was also evident in the alpha band SN strength (*p* < 0.001, Figure S1). A post-hoc analysis revealed that the SN strength decreased in the Std-condition and the Dev-condition, compared to the RS-condition (RS: 1.934 vs. Std: 1.766, *p* < 0.001; RS. 1.934 vs. Dev: 1.764, *p* < 0.001). However, there was no significant group-related effect between the MDD and HC groups.

Regardless of groups, alpha band SN strength decreased under the stimulation condition (i.e., both the Std-, and Dev-condition), suggesting that SN was well-reconfigured by the neutral-valence stimuli.


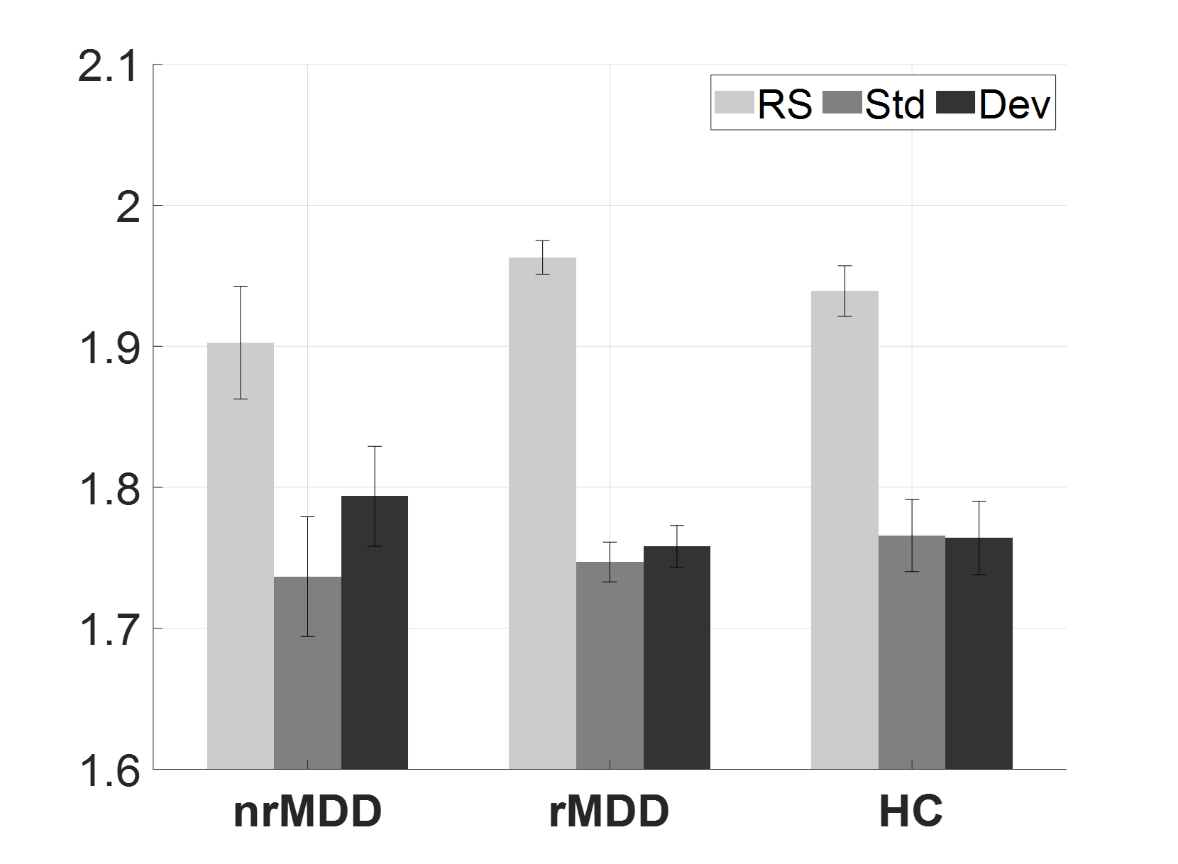


**Supplementary Figure 1.** The global strength of the alpha band salience network for each group under three different conditions.

## Total-beta band

In addition to the beta sub-bands, we further analyzed total-beta band SN (12 – 30 Hz). There was a significant group-by-condition interaction in the SN strength in the total-beta band between nrMDD and rMDD (*p* = 0.009). A post-hoc analysis revealed that nrMDD showed decreased strength in the deviant condition (0.82 vs. 0.88, *p* = 0.011; Figure S2A). Furthermore, there was a significant group-by-condition (*p* = 0.004) interaction and group effect (*p* = 0.015) in the FC between rIns and dACC. A post-hoc analysis revealed that nrMDD showed decreased FC in the deviant condition (0.26 vs. 0.30, *p* = 0.001; Figure S2B). There were no other group-related effects.

Despite the significant differences, incorporating them into the feature candidates undermined the classification performance, which is potentially due to the redundancy of the features between the high-beta and total-beta bands. Specifically, we achieved best performance using a SVM classifier (classification accuracy 77.42%, sensitivity 71.43%, specificity 82.35%) with five features. The classification performance was slightly improved by excluding potentially redundant low and high-beta band feature candidates. Finally, we achieved best performance using an LDA classifier (classification accuracy 80.65%, sensitivity 78.57%, specificity 82.35%) with three features.


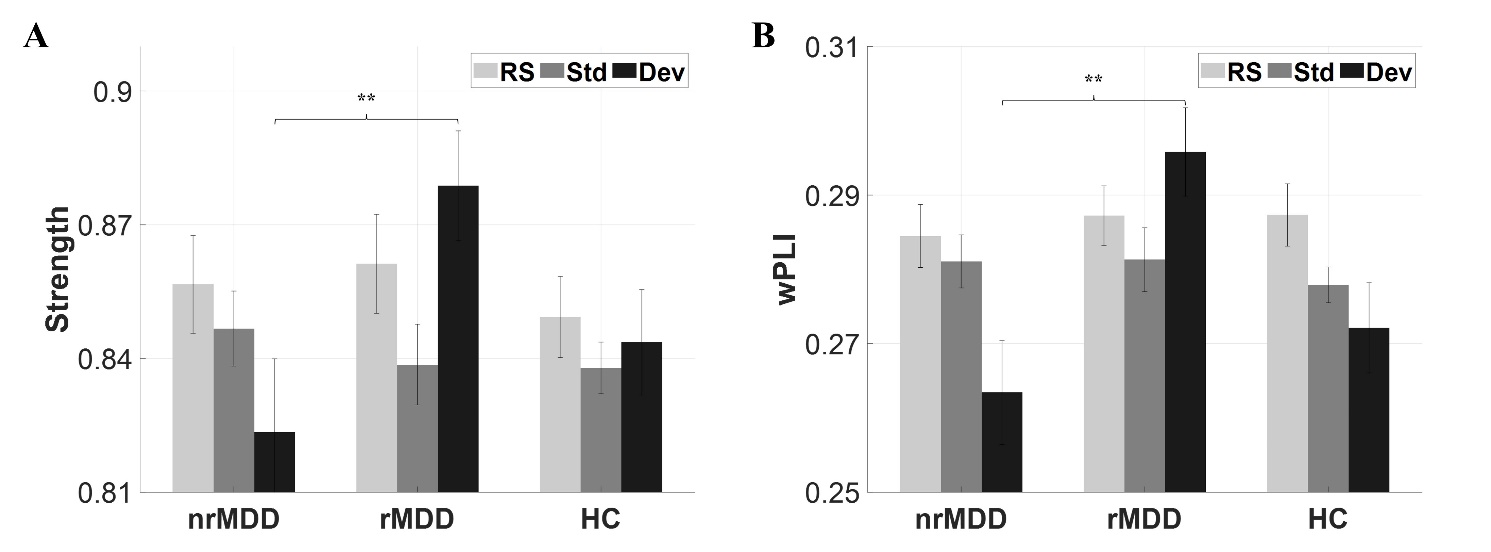


**Supplementary Figure 2.** Statistical results of the total-beta band salience network for each group under three different conditions. (A) Global strength. (B) FC. The error-bars indicate the standard errors. ** *p* < 0.01

# Correlation analysis

## Methods

To investigate the associations between the alterations in depressive symptom severity and the condition-dependent changes in FC, we performed a Pearson correlation analysis using a bootstrap resampling technique (*n* = 5,000). This approach was chosen to mitigate potential issues related to multiple corrections [1]. Specifically, the change in depressive symptom severity was quantified as the change ratio in the Ham-D scale relative to the baseline period. The condition-dependent change in FC was calculated as the difference between the FC in the Dev-condition and that in the Std-condition.

## Results

The condition-dependent change in FC between the dACC and rIns showed a negative correlation with the change ratio of the Ham-D scale at the second week in patients with MDD (r = -0.403, p = 0.025, 95%CI -0.609 ~ -0.149; Figure S3). This association suggests that the severity of depressive symptoms in patients with MDD might be sensitively relieved during early period responses to the pharmacological treatment, as the condition-dependent change in FC sensitively responds to the external stimuli.


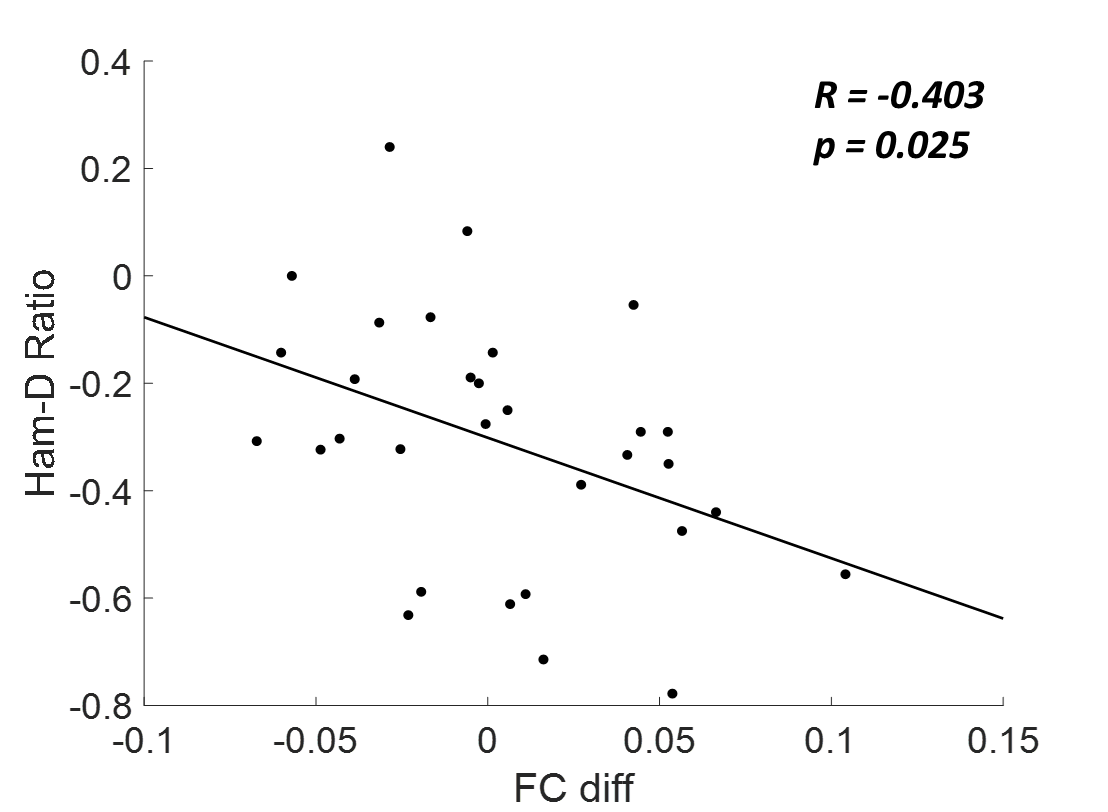


**Supplementary Figure 3.** Results of correlation analysis in patients with MDD. The difference in functional connectivity between the dACC and rIns was negatively correlated with the change ratio in the early period (i.e., 2^nd^ week) Ham-D scale in patients.

# Correlation analysis

## Regions of interests of the salience network

The ROIs of the SN were selected based on nine previous fMRI-based FBN studies that provided MNI coordinates for the ROIs [2-10]. Specifically, we included only those regions identified as nodes of the SN at least three times across these studies. Ultimately, we selected three ROIs: (i) the dorsal anterior cingulate cortex (dACC), (ii) the left insula (lIns), and (iii) the right insula (rIns). The coordinates for these ROIs were determined by identifying the center of gravity for each region (Table S2).

# Supplementary tables

**Supplementary Table 1.** Symptom severity for group across the periods

|  | **Ham-D** | | **Ham-A** | |
| --- | --- | --- | --- | --- |
| Period | nrMDD | rMDD | nrMDD | rMDD |
| Week 0 | 29.36 ± 6.18 | 26.24 ± 6.81 | 26.64 ± 6.72 | 24.76 ± 6.57 |
| Week 2 | 25.07 ± 4.89 | 14.53 ± 6.09 | 24.14 ± 4.05 | 14.94 ± 7.08 |
| Week 4 | 21.14 ± 5.64 | 9.12 ± 5.72 | 21.14 ± 5.61 | 9.18 ± 6.03 |
| Week 8 | 17.14 ± 8.05 | 4.41 ± 1.77 | 16.43 ± 7.36 | 4.06 ± 2.73 |

**Supplementary Table 2.** MNI coordinates of the ROIs for the SN

| **ROI** | **x** | **y** | **z** |
| --- | --- | --- | --- |
| dACC | 2.25 | 22.25 | 36.00 |
| lIns | -38.00 | 18.40 | -1.40 |
| rIns | 40.71 | 18.71 | -3.57 |

dACC, dorsal anterior cingulate cortex; lIns, left insula; rIns, right insula

**Supplementary Table 3.** Best classification performance for each machine learning models.

| **Model** | **Accuracy (%)** | **Sensitivity (%)** | **Specificity (%)** |
| --- | --- | --- | --- |
| LDA (n = 11) | 80.65 | 78.57 | 82.35 |
| SVM (n = 13) | 77.42 | 71.43 | 82.35 |
| KNN (n = 9, k = 3) | 67.74 | 78.57 | 58.82 |
| NB (n = 3) | 64.52 | 64.29 | 64.71 |

LDA, linear dependent analysis; SVM, support vector machine, KNN, k-nearest neighbors, NB, naïve-Bayes; n, the number of optimal feature subset; k, the number of neighbors

# Supplementary references

1. Dudoit, S., Van der Laan, M. J., & Pollard, K. S. (2004). Multiple testing. Part I. Single-step procedures for control of general type I error rates. Statistical Applications in Genetics and Molecular Biology, 3(1).
2. Brier, M. R., Thomas, J. B., Snyder, A. Z., Benzinger, T. L., Zhang, D., Raichle, M. E., ... & Ances, B. M. (2012). Loss of intranetwork and internetwork resting state functional connections with Alzheimer's disease progression. Journal of Neuroscience, 32(26), 8890-8899.
3. Uddin, L. Q., Supekar, K. S., Ryali, S., & Menon, V. (2011). Dynamic reconfiguration of structural and functional connectivity across core neurocognitive brain networks with development. Journal of Neuroscience, 31(50), 18578-18589.
4. Agosta, F., Pievani, M., Geroldi, C., Copetti, M., Frisoni, G. B., & Filippi, M. (2012). Resting state fMRI in Alzheimer's disease: beyond the default mode network. Neurobiology of aging, 33(8), 1564-1578.
5. Chen, A. C., Oathes, D. J., Chang, C., Bradley, T., Zhou, Z. W., Williams, L. M., ... & Etkin, A. (2013). Causal interactions between fronto-parietal central executive and default-mode networks in humans. Proceedings of the National Academy of Sciences, 110(49), 19944-19949.
6. Bonnelle, V., Ham, T. E., Leech, R., Kinnunen, K. M., Mehta, M. A., Greenwood, R. J., & Sharp, D. J. (2012). Salience network integrity predicts default mode network function after traumatic brain injury. Proceedings of the National Academy of Sciences, 109(12), 4690-4695.
7. Wotruba, D., Michels, L., Buechler, R., Metzler, S., Theodoridou, A., Gerstenberg, M., ... & Heekeren, K. (2014). Aberrant coupling within and across the default mode, task-positive, and salience network in subjects at risk for psychosis. Schizophrenia bulletin, 40(5), 1095-1104.
8. Krönke, K. M., Wolff, M., Shi, Y., Kräplin, A., Smolka, M. N., Bühringer, G., & Goschke, T. (2020). Functional connectivity in a triple-network saliency model is associated with real-life self-control. Neuropsychologia, 149, 107667.
9. Zheng, H., Xu, L., Xie, F., Guo, X., Zhang, J., Yao, L., & Wu, X. (2015). The altered triple networks interaction in depression under resting state based on graph theory. BioMed research international, 2015.
10. Chen, H., Li, Y., Liu, Q., Shi, Q., Wang, J., Shen, H., ... & Zhang, Y. M. (2019). Abnormal interactions of the salience network, central executive network, and default-mode network in patients with different cognitive impairment loads caused by leukoaraiosis. Frontiers in neural circuits, 13, 42.
